# Prenatal Microarray Analysis of Pregnancies Without Ultrasound Anomalies: Establishment of Copy Number and Homozygosity Frequencies in Low-Risk Population

**DOI:** 10.3390/genes17020127

**Published:** 2026-01-25

**Authors:** Stuart Schwartz, Robert G. Best

**Affiliations:** 1 Center for Molecular Biology and Pathology, Labcorp, Research Triangle Park, NC 27709, USA; bestr3@labcorp.com; 2 Department of Biomedical Sciences, University of South Carolina SOM Greenville, Greenville, SC 29605, USA

**Keywords:** single nucleotide polymorphism microarray (SNP microarray), advanced maternal age, prenatal diagnosis, low-risk population, homozygosity

## Abstract

Objectives: The overall objective of this study is to examine prenatal patients ascertained without an abnormal ultrasound (US) or an abnormal cell-free DNA (cfDNA) finding to provide a unique understanding of pathogenic copy number variants, identity by descent (IBD) and variants of uncertain significance (VUSs) in a normal population. Methods: This study retrospectively provides an analysis of over 28,362 prenatal specimens ascertained without an abnormal US or abnormal cfDNA finding utilizing an SNP microarray. These specimens include at least 10 different ascertainment groups, including advanced maternal age (AMA), anxiety, abnormal maternal serum screen (MSS) with/without AMA, and a previous or familial child/pregnancy with a chromosome abnormality or a genetic disorder. Results: This study provides a basic understanding of pathogenic copy number variants (CNVs), homozygosity and VUSs in an essentially normal population. This low-risk population has a frequency of pathogenic CNVs of ~1.26%; however, ~52% were associated with neurodevelopmental microdeletions/microduplications and ~13% were associated with incidental findings. Overall, ~1.32% of these patients showed an increase in homozygosity, the majority due to consanguinity. Lastly, VUSs were seen in 1.41% of this group, of which ~90% were familial. Conclusions: Overall, these findings provide a better estimate of the baseline frequencies and types of pathogenic CNVs and homozygosity in a low-risk population. It provides insight into the distribution of stretches of homozygosity associated with identity by descent in this population and gives a better understanding of the extent of variants of uncertain significance in phenotypically unaffected individuals.

## 1. Introduction

Morphological chromosome analysis has been the mainstay in clinical diagnostic cytogenetics laboratories since Tijo and Levan first demonstrated, in 1956, that the modal chromosome number in humans is 46 [1]. There have been many improvements of this standard methodology, including the introduction of increasingly higher-resolution chromosome analysis and fluorescence in situ hybridization to provide better resolution and detection of chromosomal abnormalities. However, it was not until 1993 that DNA was first used as an analytical tool to detect chromosomal abnormalities by comparative genomic in situ hybridization (CGH) [2,3,4]. While this technology did not necessarily improve resolution, it did lead to the development of high-resolution analysis of DNA copy number variation using CGH to microarrays (hybridization to immobilized mapped sequences instead of metaphase chromosomes) [5,6,7].

Current microarray analysis mainly focuses on improving the detection of CNVs, where alterations as low as 25 kb are routinely detected. In addition, arrays that have an SNP component can simultaneously reveal CNVs and show the presence of maternal cell admixture, chimerism, and mosaicism, as well as demonstrate identity by descent (IBD).

However, most of the array analysis is performed on a postnatal population with specific disorders (e.g., neurodevelopmental delay), a prenatal population referred because of phenotypic anomalies detected by US, or a prenatal population referred because of a suggestion of a chromosome abnormality through cfDNA studies. Most of these studies focus on copy number variation, and while there is a body of work associating CNVs with specific phenotypic anomalies, less is known about CNV findings in low-risk populations. There has been some, but limited study of fetuses whose mothers have been referred due to advanced maternal age, where the fetus has no phenotypic anomalies detected [8,9]. However, there are also other populations at low risk for CNVs including abnormal maternal serum screen (MSS), anxiety (usually <35 years of age) and families with a previous child/pregnancy with a structural/numerical chromosomal or genetic abnormality. Even less is understood about the different types of pathogenic CNVs seen in a normal population. In addition, there are scant studies on the types of homozygosity seen in normal populations and the frequency of identity by descent.

The objective of this manuscript is to interrogate and not only provide useful data describing the frequency of CNVs in a low-risk population but also characterize the types of abnormalities that occur. This is also true in examining the frequency of VUSs that are reported as well as baseline information on regions of homozygosity (ROHs) resulting from IBD. This study provides invaluable information about unaffected populations that will ultimately provide important information in understanding the affected populations that are more frequently referred for study.

## 2. Materials and Methods

Results on over 131,000 prenatal patients received for diagnostic SNP microarray testing over a 12-year period (2010–2022) were reviewed to identify results indicating pathogenic copy number, VUS, IBD, recessive genes, segmental UPD and chimerism. Clinical information was utilized in this study as provided by the referring physician.

Patients were categorized by their mode of ascertainment and all patients that were referred for specific reasons other than abnormal ultrasound findings or an abnormal cfDNA finding were included in this study. These included 28,362 patients and comprised the following: advanced maternal age (AMA; maternal age ≥ 35); anxiety (maternal age < 35, but with no other specific reason for wanting testing or carriers of a genetic mutation); abnormal maternal serum screen (MSS) including both AMA and younger patients; parents with a previously diagnosed aneuploid pregnancy; parents with a known aneuploid pregnancy or child in their family; parents with a previously diagnosed chromosomal abnormality during pregnancy or in a child; parents with a known chromosomal abnormality in their family; and parents with a previously diagnosed genetic disorder (including intellectual disability or autism) in a pregnancy or child; or parents with a known genetic disorder in their family.

### 2.1. Array Methodology

Studies were performed utilizing CytoScan^TM^ HD SNP arrays (Thermo Fisher Scientific, Waltham, MA, USA). The CytoScan^TM^ HD array contains approximately 2.695 million markers across the entire human genome. There are approximately 743,000 SNPs and 1,953,000 structural NPCN (non-polymorphic copy numbers) probes. On average, the median spacing between markers is 0.88 kb.

The CytoScan^TM^ HD Accel array contains approximately 2.772 million markers across the entire human genome. There are approximately 743,130 SNPs and 2,029,441 structural NPCNs. On average there is approximately a 0.82 kb median spacing distance between each marker. DNA was extracted utilizing standard methods and 250 ng of total genomic DNA extracted was digested with NspI and then ligated to NspI adaptors and amplified using Titanium Taq with a GeneAmp^TM^ PCR System 9700. PCR products were purified using AMPure beads and quantified using NanoDrop^TM^ 8000. Purified DNA was fragmented and biotin-labeled then hybridized to the Affymetrix Cytoscan^TM^ HD or CytoScan^TM^ HD Accel GeneChip^TM^. Data was analyzed using Chromosome Analysis Suite (ChAS 4.0 and 4.4) using the GRCh37/hg19 assembly. All data passed QC metrics for MAPD and SNP QC metrics; however, in cases where there were questions about an abnormality, confirmation testing was performed.

### 2.2. Detection of Copy Number Variants (CNVs)

Copy number variation was identified by examination of the log2 ratio, smooth signal and both B-allele and allele difference patterns. Examination of these parameters identified regions of interest at 25 kb or greater for both gains and losses. Based on the patterns, mosaicism or other admixture (of at least 10%) could also be identified. When material was available for further testing, confirmation of mosaicism was undertaken.

Pathogenic CNVs were reported if a region of >25 kb contained a deletion or entire duplication of a gene that was designated as pathogenic by either ClinGen or consensus in the literature.

Variants of uncertain significance (VUSs) were reported in prenatal studies if regions did not contain any known pathogenic genes and deletions ≥ 1 Mb and duplications ≥ 2 Mb with at least one OMIM gene present. VUS regions were also reported in smaller deletions and/or duplications in which ≥10 OMIM genes were present. It is well established that reporting criteria for prenatally detected VUSs varies considerably among laboratories in North America [10] and no specific guidelines were available at the initiation of this study. Our rationale was initially informed by our postnatal studies and the large number of familial gains and losses reported below the stated criteria. These reporting criteria are consistent with most current clinical laboratory guidelines where CNVs < 1 mb are only reported if regions are well characterized and known to be pathogenic and most laboratories usually include a size threshold of >1–3 Mb [11]. Currently ACMG/ACOG guidelines suggest not reporting small VUSs (under 500 kb deletion/1 Mb duplication) unless there is strong evidence of pathogenicity [12].

As this study took place over multiple years, it is important to note that our cut-offs did not change, but the genes of interest that were considered pathogenic increased over the years.

### 2.3. Detection of Identity by Decent (IBD)

Two types of homozygosity patterns were identified and reported in this study. The first and most prominent, consanguinity, was identified in these cases by examination of both B-allele and allele difference patterns. Contiguous homozygosity of >8 Mb within at least two chromosomes infer descent through a common ancestor. The degree of consanguinity is related to the number of homozygous regions.

In addition, a second type of identity by descent was examined in this study: a high level of allele homozygosity due to numerous contiguous short runs (associated with a geographically or socially limited gene pool). The amount of homozygosity was totaled (using all regions of homozygosity at 1 Mb or greater) and patients reported at the 99th percentile. This finding can be associated with a potential recessive allele risk.

### 2.4. Statistical Analysis

The findings from different ascertainment groups were compared using chi-square statistics with a Yates correction and a Bonferroni correction (for multiple comparisons) and were combined if they were not significantly different at the *p* < 0.001 level. However, these comparisons were complicated as there was a disparity in the overall size of different ascertainment groups.

## 3. Results

During this study, we examined over 28,362 prenatal patients in 10 different ascertainment groups that were studied for reasons other than abnormalities detected by ultrasound or abnormal prenatal cfDNA findings (Table 1). Data is presented based on the type of sample received (amniotic fluid or chorionic villus sample) and in total. Also, the types of abnormality detected by microarray analysis (not seen on chromosome analysis) are listed, whether pathogenic, incidental or a defined microdeletion/duplication that can be classified as a neurodevelopmental disorder.

### 3.1. Advanced Maternal Age

The largest ascertainment group we studied were patients referred because of AMA, where no ultrasound abnormalities were seen. This group comprised almost 60% of the patients reported in this study. Overall, there were ~1.19% pathogenic CNVs, a total of ~1.2% homozygosity findings (consanguinity or isolated population) and ~1.36% VUSs.

### 3.2. Anxiety

The second largest ascertainment group were patients referred because of pregnancy-related anxiety. Most of these patients opted for prenatal diagnosis (including microarray analysis) at age < 35. The frequency of both pathogenic CNVs and VUSs were equivalent to the AMA patients [not significant at *p* < 0.01]; however, the IBD was elevated as some of these couples may have known that they were (or might be) related.

### 3.3. Previous/Familial Aneuploid

This group consisted of couples that either had a previous aneuploid pregnancy/child or indicated that there was an aneuploid pregnancy/child in their family leading them to prenatal testing. While this referral might be associated with an increase in subsequent aneuploid pregnancies, it would not be expected to correlate with CNV abnormalities. While pathogenic VUSs were lower in the familial cases (possibly due to the low number of cases ascertained—only ~2% of total), overall, the numbers for CNVs and VUSs were comparable to the AMA findings [*p* not significant at 0.05].

### 3.4. MSS

Over 2000 patients were referred due to an abnormal MSS (or an abnormal MSS in conjunction with AMA). This includes both abnormal MSS associated with an increased risk of trisomy 18 or 21 but also those with low estriol findings and a possible association with Smith–Lemli–Opitz syndrome (SLOS). Some of these referrals have not been precisely characterized when submitted and only referred as abnormal MSS. This group has been split into two subgroups: a group referred because of a low uE3 or possible association with SLOS and a group referred because of an association with trisomy 21 or 18 or just as an abnormal MSS.

For both groups, the frequency of VUSs and consanguinity was not significantly different from the AMA population. However, the frequency of pathogenic CNVs was significantly higher (*p* < 0.0001; note that all comparisons were made with a Bonferroni correction and were only significant with *p* < 0.001 or greater), due to the association of *STS* deletions with low prenatal values of estriol [13]. In the former group (a low uE3 (Unconjugated Estriol) value or those referred to rule out SLOS because of its association with uE3), there was an obvious bias in detecting deletions of *STS*. In the latter group, there were likely some cases associated with SLOS or low uE3 not designated in the referral, which led to a higher reportable abnormal CNV frequency.

### 3.5. Previous/Familial Chromosome Abnormalities

These two groups (~2700 patients) consisted of couples that either had a previous pregnancy/child with a chromosomal abnormality or indicated that there was a pregnancy/child in their family with a chromosome abnormality (not including an aneuploid finding). While this referral might include a finding only detectable by chromosome analysis, it might also include those detected by microarray analysis but not specified in the referral. Consequently, there was both a significant increase in the frequency of pathogenic CNVs and VUSs detected. However, the frequency of homozygosity associated with IBD was similar to that found in the AMA ascertainment group.

### 3.6. Previous/Familial Genetic Disorders

The final two ascertainment groups included couples that either had a previous pregnancy/child with a genetic abnormality or indicated that there was a pregnancy/child in their family with a genetic abnormality. The groups included pregnancies/children with a specified genetic disorder, a major structural abnormality (e.g., holoprosencephaly), intellectual disability or autism. In this group the frequency of a pathogenic CNV was only slightly higher than that detected in the AMA ascertainment group and the frequency of a VUS was nearly equivalent [*p* = 0.092]. In this group, the frequency of CNVs would not be expected to be elevated as the phenotypic findings are more likely to be associated with a single-gene disease. As such, the frequency of homozygosity (indicative of IBD) was considerably higher, suggestive of the possibility of an underlying recessive disorder (from a gene in a homozygous region).

### 3.7. Pathogenic CNVs—Overall Findings

To maximize the number of pregnancies included for microarray analysis in establishing a baseline frequency of pathogenic CNVs, groups were combined based on CNV risk level. None of the groups would be expected to have an increase in the frequency of pathogenic CNVs detected by microarray analysis. These ascertainment groups included AMA, anxiety, previous aneuploid pregnancy/child and familial aneuploid pregnancy/child (Table 2; Table S1). These groups were included in the low risk level as a priori, none are known or thought to have an increased risk for a submicroscopic CNV. Overall, there were 21,401 pregnancies tested in this combined group. Abnormalities were classified as pathogenic (leading to phenotypic abnormalities detected during pregnancy or within the first two years of life), incidental (late-onset disorders such as CMT1A, HNPP and *BRCA1*) and microdeletion/duplications associated with variable neurodevelopmental disorders (NDDs, e.g., 15q11.2 and 16p11.2; Table 2). There were 270 abnormalities detected in this group (1.26%); 140 NDDs (51.9% of the total abnormalities), 35 incidental (13.0%) and 91 pathogenic (35.1%). The most common NDDs included 15q11.2 deletions and 16p13.11 duplications (Table 3). The most common incidental findings included *STS* deletions, HNPP and CMT1A (Table 4).

### 3.8. Identity by Descent (IBD)

Similarly to pathogenic CNVs, several groups were combined for analysis, none of which were expected to have an increase in the frequency of homozygosity. These ascertainment groups included AMA, previous aneuploid pregnancy/child and familial aneuploid pregnancy/child, MSS +/− AMA and MSS due to a low uE3 or R/O SLOS. Overall, there were 19,815 pregnancies tested in this group. Homozygosity was classified as either consanguinity or high-dispersed. There were 263 cases of increased homozygosity detected in this group (1.32%); 170 were associated with consanguinity (64.6% of the total) and 93 high dispersed homozygosity (35.4%) (Table 5).

### 3.9. Variants of Uncertain Significance (VUSs)

Also, like pathogenic CNVs, low-risk groups were combined for analysis, none of which were expected to have an increase in the frequency of VUSs. Low-risk ascertainment groups included most of the groups in this study: AMA, anxiety, previous pregnancy/child with a genetic abnormality and familial pregnancy/child with a genetic abnormality, previous aneuploid pregnancy/child, familial aneuploid pregnancy/child and MSS+/− AMA and MSS due to a low uE3 or R/O SLOS. Overall, there were 25,700 pregnancies examined in this combined group. VUS was defined as a CNV that was not considered pathogenic but within our reporting guidelines (see Materials and Methods). There were 361 VUSs among the combined groups (1.40%; Table 6). Parental studies were performed when samples were provided and revealed that 89.6% were familial.

## 4. Discussion

Patients are referred for prenatal diagnosis for a variety of reasons. While most are tested because of the presence of US abnormalities or because of an abnormal cfDNA test, there are many other reasons for microarray testing. These include advanced maternal age, an abnormal MSS, maternal or paternal anxiety or a previous or familial chromosomal or genetic abnormality.

Microarray analysis can detect a CNV (either a reportable abnormal CNV or VUS) as well as skewed patterns of homozygosity. Whether patients are studied using microarrays or by other molecular techniques, such as long-range sequencing, whole-genome sequencing or optical gene mapping, it is valuable to have a complete understanding of CNVs and homozygosity. The overriding purpose of this study was to use a large data set to establish a baseline distribution in low-risk populations to help to better understand the significance of CNVs and homozygosity in populations referred because of structural or developmental problems.

### 4.1. CNVs—Pathogenic

One of the goals of this study was to examine the frequency and types of pathogenic abnormalities in a large data set from a low-risk population. The low-risk population utilized to examine pathogenic CNVs was constructed using patients that were studied with indications of advanced maternal age, anxiety (usually patients < 35 wanting prenatal diagnosis), MSS+/−AMA and having a previous pregnancy/child with aneuploidy or having a family member with aneuploidy. This should provide numbers that will approximate what might be ascertained in newborns and give an understanding of both the frequencies and types of specific abnormalities. The overall frequency of microarray pathogenic CNVs was 1.26% (Table 2). These were split into three groups: pathogenic CNVs (those which would be associated with a recognizable abnormality at birth or early in life); microdeletions/duplications associated with neurodevelopmental disorders (these are highly variable disorders with reduced penetrance but are associated with the possibility of a neurodevelopmental disorder—Table 3) and incidental findings (those disorders that are more likely to be adult onset (e.g., HNPP, *BRCA1*) or do not involve intellectual or development delay (e.g., *STS*; Table 4). It is interesting to note that the NDD microdeletions/duplications comprise slightly less than 52% of the abnormalities (or 0.65% of the entire group). The most common NDD disorders detected were 16p13.11 duplications, 16p11.2 (proximal) deletions and duplications and 15q11.2 deletions (Table 3). Additionally, cancer susceptibility genes comprised 14.3% of the incidental group (Table 4). Both *ATM* and *HBB/HBD* deletions, although recessive disorders, were included in this group as carriers can manifest clinical findings. These numbers are extremely important for Genetic Counselors as the majority of prenatal patients who have diagnostic testing have microarray analysis that can reveal these NDDs and incidental disorders and this provides good baseline information to the patients.

This is not the first study to examine the frequency of CNVs in patients ascertained without US or cfDNA findings, but it is the largest to date with approximately 6 times the number of cases compared to the next largest single study and a 3-fold increase in the total number of cases with stratification by risk. In addition, we provide analysis of both aggregated homozygosity (consanguineous) and dispersed homozygosity (associated with restricted population gene flow). Part of the difficulties of many previous studies is that the papers do not always differentiate between the ascertainment groups, mixing both high-risk and low-risk populations, meaning that the existing data cannot be utilized. Table 7 lists nine previous studies (total of 10,985 patients with population sizes from 165 to 4014) of AMA ascertainment demonstrating a frequency of pathogenic CNVs of 1.31%, consistent with our findings.

In contrast to the AMA and “low-risk” population findings, ascertainment through an abnormal MSS (MSS+/− AMA and MSS due to a low uE3 or R/O SLOS) showed an increase in the frequency of microarray-detected abnormalities (2.75% and 38.60%). The latter is certainly due to the presence of *STS* deletions (22 *STS* deletions); however, the former group is also likely due to the inclusion of findings associated with an *STS* deletion. An analysis of 3490 patients from seven previous studies (Table 8) had a lower percentage of abnormal CNVs (1.81%), possibly reflecting variable inclusion of samples with low estriol values in MSS.

There is also an increase in microarray-detected abnormalities in families with a previous chromosome abnormality or with a known familial abnormality (4.87%—8.18%, Table 1). While there are several possible reasons for this elevated frequency, one is associated with mothers carrying X-chromosome deletions, as evidenced by the large number of DMD deletions detected. Additionally, in some cases there may be a subtle parental rearrangement and in other cases one parent may be unaffected but have a variable NDD with variable expressivity or reduced penetrance.

### 4.2. Regions of Homozygosity

With the utilization of SNP microarray analysis, it became apparent that homozygosity can be detected in all or part of a chromosome. This led to an understanding that IBD, including consanguinity, can be imputed from homozygosity in SNP arrays. While the exact definition varies between labs, many utilize the presence of ≥2 homozygous stretches of 8 Mb or greater on different chromosomes to denote consanguinity. It is important to consider regions of homozygosity (ROHs) associated with consanguinity, as autosomal recessive disorders occur with an increased frequency in offspring of consanguineous marriages proportional to the coefficient of relationship. When there is no known genetic disorder in the family, first-cousin marriages convey a risk for birth defects in the offspring that is approximately double the population risk [23]. Hamamy also stated that the risk may be presented as 1.7–2.8% higher than the population background risk, mostly attributable to autosomal recessive diseases. While SNP arrays cannot diagnose recessive disorders, they can provide some indication as to which genes may be implicated, based on the homozygosity seen and phenotype. However, relatively little has been published about consanguinity and homozygosity in the general population based on SNP array analysis. The low-risk population for IBD in this study was constructed using patients that were studied with indications of advanced maternal age, having a previous pregnancy/child with aneuploidy or having a family member with aneuploidy and those individuals referred because of an abnormal MSS. A priori, none of the pregnancies from these ascertainment groups are expected to have an increase in identity by descent. The overall frequency of consanguinity was 0.89% (Table 3).

A few groups were observed to have elevated frequencies of consanguinity in our study (Table 1). Patients ascertained because of parental anxiety had a frequency of consanguinity of 2.5% [*p* < 0.00001]. This is likely because patients less than 35 years of age may seek out prenatal diagnosis because of a known/ possible relationship with their partner. However, the highest frequency of consanguinity was in groups ascertained because of a previous child/pregnancy with a genetic disorder or with a family member/pregnancy with a genetic disorder. The frequencies of consanguinity in these two groups were 4.66% and 3.28%, respectively, numbers that are significantly higher than in the combined low-risk population (both *p* < 0.00001). These numbers may suggest autosomal recessive disorders in these families consequent to inheritance from a common ancestor.

### 4.3. High Dispersed Homozygosity/IBD

Although elevated homozygosity is most often associated with consanguinity, a second category showing an increase in small regions of homozygosity is associated with an isolated population. This was best described by [24], who stated that these small, isolated communities had an increased number of ROHs from 0.5 to 2.0 Mb, resulting in very high frequencies of homozygosity. The higher the number of short homozygous segments, the higher the probability that the parents might be from an isolated population. It is well established that individuals from less-outbred communities have an increase in recessive diseases. Examples include Tay–Sachs Disease, which is more common in people of Ashkenazi Jewish heritage, and Batten Disease, which is more common in the Shetland Islands [25], along with several diseases (e.g., Ellis-van-Creveld syndrome and maple syrup urine disease) in the Amish population [26].

The overall frequency of high dispersed homozygosity associated with the possibility of an isolated population was 0.55% in our low-risk population using a 99th percentile threshold (Table 5). However, we observed one group with significantly elevated frequencies of high dispersed homozygosity (Table 1): patients ascertained because of a previous child/pregnancy with a genetic disorder demonstrated a frequency of 1.77% (*p* < 0.00001). This number is suggestive that some of the genetic disorders may be consistent with autosomal recessive disease in these families as a result of being in an isolated population.

High dispersed homozygosity from clinical prenatal SNP array studies has not been extensively studied and seems to merit further attention. From the standpoint of clinical actionability, high levels of homozygosity in families who do not meet criteria for consanguinity may benefit from the availability of carrier screening for recessive disorders. In addition, pregnancies with a known phenotype associated with various AR genes may benefit from a focused study for single nucleotide variants when there is clear evidence of IBD in a region of clinical significance.

### 4.4. Variants of Uncertain Significance (VUSs)

One constant feature in diagnostic testing for CNVs is variants whose significance is not well understood. Based on our reporting definitions for VUSs, the overall frequency in the low-risk population was 1.41% (Table 4). Additionally, we determined that ~90% of all the VUSs detected in this population were familial when parental follow-up was available (in 87% of studies), suggesting that the vast majority of these are not clinically significant.

The frequency of VUSs was similar for almost all of the ascertainment groups with the exception of cases ascertained with a previous chromosomal abnormality (6.42%; *p* < 0.00001) or familial chromosome abnormality (5.22%; *p* < 0.0001). These frequencies are probably elevated as parents were likely carriers of a VUS, reinforcing the need that it is not necessary to follow these patients.

Overall, re-classification of genes detected in microarray analysis is problematic and, in the context of this study, is probably most applicable to those cases where the gain or loss is de novo. These families are initially informed that there is an increased risk of clinical abnormalities and the significance of the CNV will always be re-examined.

### 4.5. Limitations and Strengths of the Study

Although all patients studied were provided with an indication for study, in some cases a specific genetic disease or previous chromosome abnormality was not provided, limiting the ability to correlate our findings. As the patients were referred from multiple sources, the authors were dependent on the referring diagnosis and classification. As this study included data from over 12 years, some of the reporting criteria changed over time (e.g., 15q11.2 deletions were not initially reported). However, these limitations involved all of the groups and with a study of this magnitude, our underlying questions could be answered.

The review of the literature was hampered as many individual studies did not separate out their findings between AMA and abnormal US populations and this study allows a much greater population to understand all of these frequencies.

## 5. Conclusions

The major objective of this study was to obtain baseline information for pathogenic CNVs, homozygosity and VUSs in a low-risk prenatal population, providing a stronger foundation for genetic counseling in both low- and high-risk pregnancies.

The overall frequency of pathogenic microarray CNVs in low-risk pregnancies was 1.24%. Approximately 53% of these abnormalities were NDD microdeletions/microduplications and another 13% were incidental findings. Although some of these may have no discernible clinical consequence on the fetus, they are important to detect and report for the purposes of family decision-making. This potentially has a significant effect on reproductive and health planning for the entire family. However, the reporting of the incidental findings must consider both the potential benefits and harm before reporting and the specific findings in each case must be considered [27]. The reporting of incidental findings was performed according to the laboratory policy in place at the time of testing. These findings will ultimately help to correlate any unusual US findings with specific NDDs and clarify the contrast in risk between baseline and higher-risk populations as the field continues to progress.

Homozygosity associated with consanguinity and isolated populations was not prevalent in our low-risk population and was relatively unlikely to be associated with poor clinical outcomes (0.89% and 0.55%, respectively). However, ROHs were significantly elevated in families with previous genetic disorders, suggesting the potential for SNP microarray to unmask cases or the risk of recessive disease that might be addressed through molecular studies for SNV or carrier screening.

Variants of uncertain significance are unavoidable in high-complexity clinical genomic testing. In our study, the baseline frequency was 1.40% among results provided to patients. Reassuringly, nearly 90% were familial, indicating a low overall probability of poor clinical outcomes in these cases. Comparison of these findings to those found in the abnormal ultrasound population will provide a better overall understanding of these variants.

## Figures and Tables

**Table 1:** This table shows the 10 ascertainment groups in this study along with the frequency of pathogenic CNVs, homozygosity and VUSs.

			Reportable Abnormal CNVs				Homozygosity		VUS	
Ascertainment	Specimen	Total	Pathogenic	Incidental	Micro Del-Dup	Total	Consanguinity	Highly Dispersed	VUS	% De Novo
AMA	Amnio	9980	46	12	72	130 (1.30%)	89	61	144 (1.44%)	11.9%
	CVS	6326	22	12	30	64 (1.01%)	37	9	80 (1.26%)	12.9%
	Total	16,306	68	24	102	194 (1.19%)	126 (0.77%)	70 (0.43%)	222 (1.36%)	12.1%
Anxiety	Amnio	2369	15	8	23	46 (1.94%)	57	17	31 (1.30%)	3.3%
	CVS	1344	6	1	7	13 (0.97%)	36	1	21 (1.56%)	15.0%
	Total	3713	21	9	30	59 (1.59%)	93 (2.50%)	18 (0.48%)	52 (1.40%)	8.0%
Previous— Aneuploid	Amnio	522	5	1	4	10 (1.81%)	8	8	7 (1.33%)	0.0%
	CVS	581	2	1	3	6 (1.03%)	5	2	8 (1.37%)	0.0%
	Total	1103	7	2	7	16 (1.45%)	13 (1.18%)	10 (0.91%)	15 (1.36%)	0.0%
Familial— Aneuploid	Amnio	157	0	0	1	1 (0.64%)	3	3	1 (0.64%)	0.0%
	CVS	122	0	0	0	0	1	1	0	0.0%
	Total	279	0	0	1	1 (0.36%)	4 (1.43%)	4 (1.43%)	1 (0.36%)	0.0%
LOW UE3 Or R/O SLOS	Amnio	57	0	17	0	22 (38.60%)	1	0	0	N/A
	CVS	N/A	N/A	N/A	N/A	N/A	N/A	N/A	N/A	N/A
	Total	57	0	17	0	22 (38.60%)	1 (1.75%)	1 (0.0%)	0	N/A
MSS +/− AMA	Amnio	1826	15	11	23	49 (2.68%)	23	7	28 (1.53%)	0.0%
	CVS	244	2	0	6	8 (3.28%)	3	1	3 (1.23%)	0.0%
	Total	2070	16	11	29	57 (2.75%)	26 (1.26%)	8 (0.39%)	31 (1.50%)	0.0%
Previous—Chromosome	Amnio	675	9	6	18	33 (4.89%)	10	11	51 (7.56%)	4.1%
	CVS	618	11	0	19	30 (4.85%)	8	4	32 (5.18%)	9.7%
	Total	1293	20	6	37	63 (4.87%)	18 (1.39%)	15 (1.16%)	83 (6.42%)	6.5%
Previous— Genetic	Amnio	658	5	4	11	20 (3.04%)	29	15	10 (1.52%)	0.0%
	CVS	415	0	0	3	3 (0.72%)	21	4	11 (2.65%)	0.0%
	Total	1073	5	4	14	23 (2.14%)	50 (4.66%)	19 (1.77%)	21 (1.96%)	0.0%
Familial—Chromosome	Amnio	814	46	14	14	74 (9.09%)	5	8	41 (5.07%)	2.6%
	CVS	609	26	2	19	47 (7.72%)	4	2	33 (5.41%)	0.0%
	Total	1423	69	12	33	121 (8.50%)	9 (0.63%)	10 (0.70%)	74 (5.20%)	1.4%
Familial— Genetic	Amnio	681	3	3	6	12(1.76%)	20	9	15 (2.21%)	14.3%
	CVS	418	4	2	2	8 (1.91%)	16	0	4 (0.96%)	0.0%
	Total	1099	7	4	8	20 (1.82%)	36 (3.28%)	9 (0.82%)	19 (1.73%)	9.1%

**Table 2:** This table lists the four ascertainment groups that can be utilized to determine the frequency of overall pathogenic CNVs, including pathogenic, incidental and microdeletions/microduplications. It shows the total number of each type of abnormality and the total numbers from each ascertainment group.

		Abnormalities			
Ascertainment	Total	Pathogenic	Incidental	Micro Del-Dup	Total
AMA	16,306	68	24	102	194 (1.19%)
Anxiety	3713	20	9	30	59 (1.59%)
Previous—Aneuploid	1103	7	2	7	16 (1.45%)
Familial—Aneuploid	279	0	0	1	1 (0.36%)
	21,401	95	35	140	270 (1.26%)

**Table 3:** This table shows the different microdeletions/duplications seen in the four ascertainment groups included. The NDD abnormalities make up 52.6% of all of the pathogenic abnormalities seen in this group. The most common findings include 16p13.11 microduplications, 15q11.2 microdeletions, 16p11.2 distal microdeletions and 16p11.2 proximal microdeletions and microduplications.

Type	AMA	Anxiety	Previous Aneuploid	Familial Aneuploid	Total
1q21.1—Deletion	6	2			8
1q21.1—Duplication	1	2			3
2p16.3 (*NRXN1*)—Deletion	1	5			6
2q13—Deletion	1	2	1		4
2q13—Duplication	1				1
3q28-q29—Deletion	1				1
3q28-q29—Duplication	1				1
15q11.2—Deletion	7	5			12
15q11.2-q13 Duplication	1				1
15q13.2-q13.3—Deletion	5				5
15q13.2-q13.3—Duplication	4	1			5
16p13.11—Deletion	5	1	1		7
16p13.11—Duplication	22	4	1		27
16p12.1—Deletion	4				4
16p11.2—Deletion (Distal)	2				2
16p11.2—Deletion (Proximal)	7	3	1	1	12
16p11.2—Duplication (Proximal)	10	2	1		13
VCF—Duplication	12	2			14
22q11.2 (C-D)—Deletion	5		1		6
22q11.2 (C-D)—Duplication	3				3
22q11.2 (D-F/H)—Deletion	2	1			3
22q11.2 (D-F/H)—Duplication	1		1		2
	102	30	7	1	140

**Table 4:** This table shows the different incidental abnormalities seen in the four ascertainment groups included. The incidental abnormalities make up 15.8% of all of the pathogenic abnormalities seen in this group. The most common findings include *STS* deletions and HNPP microduplications.

Type	AMA	Anxiety	Previous Aneuploid	Familial Aneuploid	Total
CMT1A	2	1			3
HNPP	6	4			10
*STS* Deletion	9	2			11
*HBB*/*HBD* Deletion	3	1	2		6
*PMS2* Deletion	2				2
*ATM* Deletion	1	1			2
*BARD* Deletion	1				1
	24	9	2	0	35

**Table 5:** This table lists the five ascertainment groups that can be utilized to determine the frequency of overall findings associated with an increase in homozygosity. It shows the total number of each type of homozygosity increase and the percentage of each type.

		Homozygosity	
Ascertainment	Total	Consanguinity	High Dispersed
AMA	16,306	126 (0.78%)	70 (0.43%)
Previous—Aneuploid	1103	13 (1.18%)	10 (0.90%)
Familial—Aneuploid	279	4 (1.43%)	4 (1.43%)
MSS (Low uE3 or R/O SLOS)	57	1 (1.92%)	1 (0.0%)
MSS +/− AMA	2070	26 (1.26%)	8 (0.39%)
TOTAL	19,815	170 (0.86)	93 (0.46%)

**Table 6:** This table lists the eight ascertainment groups that can be utilized to determine the frequency of overall VUSs. It shows the total number of each type of VUS and the percentage of these VUSs that are de novo.

		VUS	
Ascertainment	Total	VUS	% De Novo
AMA	16,306	222 (1.36%)	12.12%
Anxiety	3713	52 (1.40%)	8.00%
Previous—Aneuploid	1103	15 (1.36%)	0.0%
Familial—Aneuploid	279	1 (0.36%)	0.0%
Low uE3 or R/O SLOS	57	0 (1.49%)	N/A
MSS +/− AMA	2070	31 (1.50%)	0.0%
Previous—Genetic	1073	21 (1.96%)	0.0%
Familial—Genetic	1099	19 (1.73%)	9.09%
	25,700	361 (1.40%)	10.42%

**Table 7:** This table lists nine previous studies that show the frequency of pathogenic CNVs in an AMA population. It shows the total number of patients studied, the number of VUSs and the percentage in each study.

Reference	Total	Pathogenic CNV	Percent
Wapner et al. (2012) [8]	1966	34	1.73%
Armengol et al. (2012) [14]	165	3	1.82%
Fiorentino et al. (2011, 2013) [15,16]	1084	6	0.55%
Oneda et al. (2014) [17]	187	7	3.74%
Wang et al. (2018) [18]	1233	15	1.22%
Wu et al. (2020) [19]	420	17	0.95%
Xiang et al. (2020) [20]	1710	29	1.70%
Maya et al. (2021) [9]	4014	29	0.72%
Cao et al. (2023) [21]	206	4	1.94%
	10,985	144	1.31%

**Table 8:** This table lists seven previous studies that show the frequency of pathogenic CNVs in a population ascertained because of an abnormal MSS. It shows the total number of patients studied, the number of VUSs and the percentage in each study.

Reference	Total	Pathogenic CNV	Percent
Wapner et al. (2012) [8]	729	12	1.65%
Arrmengol et al. (2012) [14]	224	1	0.45%
Fiorentino et al. (2013) [16]	26	0	0.00%
Oneda et al. (2014) [17]	86	0	0.00%
Wang et al. (2018) [18]	1039	17	1.64%
Sagi-Dain et al. (2019) [22]	207	2	0.97%
Xiang et al. (2020) [20]	1179	31	2.63%
	3490	63	1.81%

## Data Availability

Data is available on request from the authors. Please contact Stuart Schwartz (schwas1@labcorp.com).

## Supplementary Materials

The following supporting information can be downloaded at https://www.mdpi.com/article/10.3390/genes17020127/s1, Table S1: Breakpoints for the pathogenic CNVs.

## Abbreviations

US	Ultrasound
cfDNA	Cell-free DNA
CNV	Copy number variant
IBD	Identity by decent
VUS	Variant of uncertain significance
AMA	Advanced maternal age
MSS	Maternal serum screen
CGH	Comparative genomic in situ hybridization
ROH	Region of homozygosity
UPD	Uniparental disomy
NPCN	Non-polymorphic copy number
SLOS	Smith–Lemli–Opitz syndrome
NDD	Neurodevelopmental disorder
