# Supplementary material for: Prenatal Microarray Analysis of Pregnancies Without Ultrasound Anomalies: Establishment of Copy Number and Homozygosity Frequencies in Low-Risk Population
Source: Genes (Basel). 2026 Jan 25;17(2):127. doi: 10.3390/genes17020127 (PMC12941352; doi:10.3390/genes17020127)
Supplement: Supplementary file 1 [file genes-17-00127-s001.zip › genes-3890068-supplementary.pdf]

Table S1: Breakpoints for the pathogenic CNVs

ASCERTAINMENT	CYTOGENETIC RESULT	MICROARRAY RESULT	BREAKPOINTS
AMA - AMNIO	46,XY	210 KB INTERSTITIAL DELETION OF 7Q11.22->Q11.22	7q11.22(70,180,371-70,390,176)x1
	46,XX	48 KB INTERSTITIAL DELETION OF 7Q11.22->7Q11.22	7q11.22(70,188,374-70,236,724)x1
	46,XY	107 KB INTERSTITIAL DUPLICATION OF 2P16.3->P16.3	2p16.3(50,540,644-50,647,947)x3
	46,XY	208 KB INTERSTITIAL DELETION OF 2P16.3->P16.3	2p16.3(51,008,639-51,216,518)x1
	46,XY	259 KB INTERSTITIAL DELETION OF 2P16.3->2P16.3	2p16.3(50,975,431-51,234,040)x1
	46,XY	312 KB INTERSTITIAL DELETION OF 2P16.3->P16.3	2p16.3(51,008,639-51,320,490)x1
	46,XX	685 KB INTERSTITIAL DELETION OF 2P16.3->2P16.3	2p16.3(50,977,215-51,662,251)x1
	46,XY	75 KB INTERSTITIAL DELETION OF 2P16.3->2P16.3	2p16.3(51,078,335-51,153,560)x1
	46,XX	87 KB INTERSTITIAL DUPLICATION OF 2P16.3->P16.3	2p16.3(50,739,312-50,826,550)x3
	46,XY	1.0 MB TERMINAL DELETION OF XPTER/YPTER->XP22.33/YP11.32	Xp22.33 or Yp11.32(168,546-1,001,584 or 118,546-951,584)x1
	46,XY	1.00 MB MOSAIC INTERSTITIAL DELETION OF YQ11.221->Q11.222;<br>7.76 MB INTERSTITIAL DELETION OF YQ11.222->Q11.23	Yq11.221q11.222(19,585,046-20,586,110)x0~1,<br>Yq11.222q11.23(20,586,246-28,341,390)x0
	46,XX	1.13 MB TERMINAL DELETION OF 12PTER->12P13.33;<br>281 KB TERMINAL DUPLICATION OF 22Q13.33->22QTER	12p13.33(173,786-1,301,401)x1,<br>22q13.33(50,916,642-51,197,838)x3
	ND	1.59 MB INTERSTITIAL DELETION OF 17Q12->Q12	17q12(34,822,465-36,410,559)x1
	46,XX	1.59 MB TERMINAL DELETION OF 12P13.33->P13.33	12p13.33(173,786-1,766,435)x1
	46,XX	1.8 MB TERMINAL DELETION OF XQ28->Q28	Xq28(153,429,330-155,233,731)x1
	ND	1.91 MB INTERSTITIAL DUPLICATION OF XQ28->Q28	Xq28(151,841,205-153,749,957)x2
	46,XX	11.2 MB INTERSTITIAL DELETION OF XP11.4->P11.22	Xp11.4p11.22(40,162,616-51,353,865)x1
	46,XY	13.7 MB TERMINAL MOSAIC GAIN OF 18P11.32->18P11.21	18p11.32p11.21(136,226-13,852,488)x2-4
	46,XY	13.8 MB MOSAIC TERMINAL GAIN OF 12P13.33->12P13.1;<br>20.8 MB MOSAIC INTERSTITIAL GAIN OF 12P13.1->12P11.1;<br>68 KB INTERSTITIAL DUPLICATION OF XP21.1->XP21.1	12p13.33p13.1(173,786-14,019,157)x2~4,<br>12p13.1p11.1(14,020,249-34,835,837)x2~6,<br>Xp21.2p21.1(31,489,959-31,558,340)x2
	46,XX	170 KB INTERSTITIAL DUPLICATION OF XP21.1->P21.1	Xp21.1(32,408,672-32,578,578)x3

	46,XY	189 KB INTERSTITIAL DELETION OF 15Q26.3->Q26.3	15q26.3(99,107,221-99,295,926)x1
	46,XY	194 KB INTERSTITIAL DELETION OF 1Q21.3->Q21.3;<br>273 KB INTERSTITIAL DELETION OF XP21.1->P21.1	1q21.3(152,183,585-152,377,818)x1,<br>Xp21.1(32,518,576-32,791,880)x0
	46,XX	2.82 MB INTERSTITIAL DELETION OF 9Q22.32->Q22.33	9q22.32q22.33(96,969,363-99,789,002)x1
	46,XY	26.32 MB MOSAIC TERMINAL DUPLICATION OF 4Q32.3->QTER;<br>4.18 MB MOSAIC TERMINAL DELETION OF 14Q32.32-QTER	4q32.3q35.2(164,834,331-190,957,473)x2~3,<br>14q32.32q32.33(103,098,835-107,285,437)x1~2
	46,XY	32.51 MB MOSAIC SEGMENTAL UPD OF 11PTER->P13	11p15.5p13(198,510-32,508,526)x2 hmz(41%)
	46,XY	355 KB INTERSTITIAL DUPLICATION OF 17P13.3->P13.3	17p13.3(1,024,520-1,379,987)x3
	46,XX	361 KB INTERSTITIAL TRIPLICATION OF XQ28->XQ28	Xq28(153,200,050-153,560,681)x4
	46,XX	4.0 MB TERMINAL DUPLICATION MOSAICISM 5Q35.3->Q35.3 ;<br>5.3 MB TERMINAL DELETION MOSAICISM 10Q26.2->Q26.3	5q35.3q35.3(176,652,863-180,648,971)x2~3<br>10q26.2q26.3(128,818,060-134,130,974)x1~2
	46,XY	4.47 MB TERMINAL DELETION OF 5Q35.2->Q35.3<br>8.68 MB TERMINAL DUPLICATION OF 21Q22.13->Q22.3	5q35.2q35.3(176,249,108-180,719,789)x1<br>21q22.13q22.3(39,421,573-48,097,372)x3
	46,XY	438 KB TERMINAL DELETION OF 16PTER->16P13.3	16p13.3(96,986-438,517)x1
	46,XX	445 KB INTERSTITIAL DELETION OF XQ28->Q28	Xq28(154,120,620-154,565,718)x1
	46,XX	445 KB INTERSTITIAL DELETION OF XQ28->Q28	Xq28(154,120,620-154,565,718)x1
	ND	445 KB INTERSTITIAL DUPLICATION OF XQ28->Q28	Xq28(154,120,599-154,565,718)x1
	46,XX	6.0 MB DELETION OF 15Q11.2->Q13.1	15q11.2q13.1(22,770,421-28,823,722)x1
	ND	6.24 MB TERMINAL DELETION OF 15Q11.2->15Q13.1;<br>565 KB INTERSTITIAL DUPLICATION OF 21Q22.2->Q22.2	15q11.2q13.1(22,770,421-29,013,164)x1,<br>21q22.2(40,847,895-41,412,643)x3
	46,XY	6.8 MB INTERSTITITAL DELETION OF 7P14.3->P14.2	7p14.3p14.2(29,072,628-35,911,739)x1
	46,XY	65 KB INTERSTITIAL DUPLICATION OF XP22.2->P22.2	Xp22.2(10,561,020-10,625,811)x2
	46,XX	7.4 MB INTERSTITIAL DELETION 10Q22.3->10Q23.2	10q22.3q23.2(81,603,169-89,017,042)x1
	46,XX	7.54 MB MOSAIC DELETION OF 21Q22.2->21QTER	21q22.2q22.3(40,580,258-48,129,894)x1~2
	46,XX	79 KB INTERSTITIAL DELETION AT XP21.1->P21.1	Xp21.1(31,734,779-31,814,158)x1
	46,XY	8.6 MB MOSAIC INTERSTITIAL DUPLICATION OF 8P23.2->8P23.1	8p23.2p23.1(3,277,855-11,858,320)x2~3
	46,XX	932 KB INTERSTITIAL DUPLICATION OF 6P21.1->P21.1	6p21.1(45,221,758-46,153,323)x3

	46,XX	980 KB INTERSTITIAL DELETION OF 22Q12.1->Q12.1;<br>4.58 MB INTERSTITIAL DUPLICATION OF 22Q12.1->Q12.3;<br>1.09 MB INTERSTITIAL DELETION OF 22Q12.3->Q12.3;<br>481 KB INTERSTITIAL DELETION OF 22Q13.1->Q13.2;<br>2.64 MB TERMINAL DELETION OF 22Q13.32->Q13.33	22q12.1(26,588,744-27,569,236)x1,<br>22q12.1q12.3(27,958,781-32,540,655)x3,<br>22q12.3(33,204,716-34,294,919)x1,<br>22q13.1q13.2(40,721,102-41,201,691)x1,<br>22q13.32q13.33(48,556,939-51,197,838)x1
	46,XX	~30.0% MOSAICISM FOR 11PTER->P15.1 ALLELE HOMOZYGOSITY	11p15.5p15.1(210,898-16,422,468)x2 hmz
	46,XX	~60.0% MOSAICISM FOR 11PTER->P15.1 ALLELE HOMOZYGOSITY	11p15.5p15.4(198,509-9,255,996)x2 hmz
	46,XX	50.30 MB MOSAIC INTERSTITIAL DELETION OF XP22.3->P11.22;<br>52.95 MB MOSAIC INTERSTITIAL DUPLICATION OF XP11.22->Q21.33;<br>60.55 MB INTERSTITIAL DELETION OF XQ21.33->Q28	Xp22.3p11.22(168,547-50,465,744)x~1.4,<br>Xp11.22q21.33(50,465,745-93,413,620)x~1.8,<br>Xq21.33q28(93,413,621-153,963,394)x~1.4
AMA - CVS	46,XY	64 KB INTERSTITIAL DELETION OF 7Q11.22->7Q11.22	7q11.22(69,735,495-69,799,959)x1
	46,XY	25.9 MB MOSAIC TERMINAL GAIN OF 20PTER->P11.1	20p13p11.1(61,568-25,898,878)x2-3
	46,XY	78.2 MB MOSAIC TERMINAL DUPLICATION OF 8Q13.2->Q24.3	8q13.2q24.3(68,147,753-146,364,022)x2~3
	46,XY	6.10 MB MOSAIC INTERSTITIAL DELETION OF 11P15.5->P15.4	11p15.5p15.4(1,621,232-7,721,870)x1~2
	ND	249 KB INTERSTITIAL DELETION OF 2P16.3->P16.3	2p16.3(50,899,536-51,149,017)x1
	46,XY	139 KB INTERSTITIAL DELETION OF 2P16.3->P16.3	2p16.3(51,067,174-51,206,342)x1
	46,XY	1.15 MB INTERSTITIAL DUPLICATION OF 11P15.5->11P15.5	11p15.5(1,625,614-2,778,506)x3
	46,XX	3.07 MB DUPLICATION OF 8P23.1P22	8p23.1p22(9,710,942-12,777,828)x3
	46,XX	49.1 MB MOSAIC GAIN OF 11Q14.2->QTER	11q14.2q25(18,061,082-106,360,585)x2~3
	46,XX	12.61 MB INTERSTITIAL DUPLICATION OF XQ22.1->Q23	Xq22.1q23(102,342,939-114,956,156)x3
	46,XY	6.0 MB INTERSTITIAL DELETION 15Q11.2->15Q13.1	15q11.2q13.1(22,770,421-28,828,168)x1
	46,XY	793 KB INTERSTITIAL DELETION OF XP22.33->XP22.33,<br>OR YP11.32->YP11.32	Xp22.33(565,806-1,358,900)x1<br>or Yp11.32(480,573-852,624)x1
	ND	1.03 MB INTERSTITIAL DELETION OF 4Q31.22->Q31.23	4q31.22q31.23(148,011,767-149,042,777)x1
	46,XX	83.8 MB TERMINAL DUPLICATION OF 11P11.12->Q25	11p11.12q25(51,164,493-134,938,470)x3
	46,XY	1.22 MB INTERSTITIAL DUPLICATION OF 5P13.3->P13.2	5p13.3p13.2(32,903,508-34,124,081)x3
	ND	7.31 MB INTERSTITIAL DUPLICATION OF 10Q22.3->Q23.2	10q22.3q23.2(81,644,079-88,957,342)x3
	ND	763 KB INTERSTITIAL DUPLICATION OF 17P13.3->P13.3	17p13.3(2,306,057-3,069,287)x3
	46,XY	329 KB DUPLICATION OF Xp21.1->Xp21.1	Xp21.1(33,078,993-33,407,744)x2
	46,XX	221 KB INTERSTITIAL DUPLICATION 6P25.3->P25.3	6p25.3(1,556,292-1,777,102)x3

	46,XY	12.9 MB TERMINAL DUPLICATION OF 20Q13.2->20QTER	20q13.2q13.33(49,928,419-62,915,555)x3
	46,XX	9.2 MB TERMINAL DELETION 10Q26.13->QTER	10q26.13q26.3(126,338,092-135,427,143)x1
	ND	445 KB INTERSTITIAL DELETION OF XQ28->Q28	Xq28(154,120,620-154,565,718)x1
ANXIETY - AMNIO	46,XY	184 KB INTERSTITIAL DELETION OF 2P16.3->P16.3	2p16.3(51,120,555-51,304,763)x1
	ND	270 KB INTERSTITIAL DELETION OF 2P16.3->2P16.3	2p16.3(51,031,691-51,301,447)x1
	46,XX	293 KB INTERSTITIAL DELETION OF 2P16.3->2P16.3	2p16.3(51,089,735-51,382,914)x1
	46,XX	94 KB INTERSTITIAL DELETION 2P16.3->2P16.3	2p16.3(51,099,074-51,193,164)x1
	ND	540 KB INTERSTITIAL DELETION OF 17Q21.31->17Q21.31	17q21.31(43,688,308-44,228,770)x1
	ND	1.67 MB INTERSTITIAL DELETION 7Q11.23->7Q11.23	7q11.23(72,621,462-74,297,923)x1
	46,XY	1.03 MB MOSAIC INTERSTITIAL DELETION OF YQ11.221->Q11.222;<br>5.66 MB INTERSTITIAL DELETION OF YQ11.222->Q11.23	Yq11.221q11.222(19,585,047-20,614,829)x0.5,<br>Yq11.222q11.23(20,614,829-26,273,936)x0
	ND	7.04 MB TERMINAL DELETION OF 17PTER->17P13.1	17p13.3p13.1(525-7,037,123)x1
	46,XX	5.04 MB INTERSTITIAL DELETION OF 15Q11.2->15Q13.1	15q11.2q13.1(23,615,768-28,659,911)x1
	46,XX	445 KB INTERSTITIAL DELETION OF XQ28->XQ28	Xq28(154,120,620-154,565,718)x1
	46,XX	1.4 MB TERMINAL DELETION OF 10Q26.3->QTER	10q26.3(134,007,160-135,427,143)x1
	46,XX	49 KB INTERSTITIAL DELETION OF 15Q23->Q23	15q23(67,528,749-67,577,560)x1
	46,XX	613 KB INTERSTITIAL DUPLICATION OF 2Q24.3->Q24.3	2q24.3(165,631,337-166,243,837)x3
	46,XY	3.95 MB SEGMENTAL UPD (4 MB - ~30%) 11P15.5 -> P11.2	11p15.5p11.2(210,898-3,951,043)x2 hmz (~30%)
	46,XX	895 KB INTERSTITIAL DUPLICATION OF PAR1 XP22.33->P22.33 OR YP11.32->P11.31;<br>2.83 MB INTERSTITIAL GAIN OF YP11.31->P11.2	Xp22.33 or Yp11.32p11.31(1,801,367-2,696,690 or 1,751,367-2,646,690)x3<br>Yp11.31p11.2(2,650,140-5,483,659)x1
ANXIETY - CVS	46,XY	4.06 MB INTERSTITIAL DELETION OF XP21.1->P11.4	Xp21.1p11.4(34,518,623-3,858,301)x0
	46,XX[29]/46,XY[4]	21.0 MB TERMINAL MOSAIC DUPLICATION OF 3P26.3->P24.3 (MCC)	3p26.3p24.3(1-21,020,874)x2.2
	46,XY	MOSAIC 6.64 MB INTERSTITIAL DUPLICATION OF XQ27.3->Q28	Xq27.3q28(147,011,698-153,653,607)x1~2
	46,XX	5.63 MB INTERSTITIAL DELETION AT 10Q11.22->Q11.23	10q11.22q11.23(46,246,711-51,874,356)x1
	46,XY	6.2 MB INTERSTITIAL DELETION OF YQ11.221->Q11.223	Yq11.221q11.223(19,574,920-25,849,444)x0
	46,XY	4.56 MB INTERSTITIAL DELETION AT 3Q11.2->Q11.2	3q11.2q11.2(93,634,519-98,194,372)x1
PREVIOUS - ANEUPLOID/AMNIO	46,XY	48 KB INTERSTITIAL DELETION AT 7Q11.22->Q11.22	7q11.22(70,188,374-70,236,724)x1

	46,XY	477 KB INTERSTITIAL DUPLICATION OF XP22.12->XP22.12	Xp22.12(20,184,364-20,661,702)x3
	46,XX	2.21 MB INTERSTITIAL DELETION OF 5Q35.2->Q35.3	5q35.2q35.3(175,188,096-177,401,384)x1
	ND	8.15 MB INTERSTITIAL DELETION OF XP22.1->11.4	Xp21.1p11.4(31,595,497-39,752,020)x1
	46,XX	13.73 MB INTERSTITIAL DELETION OF 2P16.1->P13.3	2p16.1p13.3(56,098,568-69,824,632)x1
PREVIOUS - ANEUPLOID/CVS	ND	3.47 MB INTERSTITIAL DUPLICATION OF 17P11.2->P11.2	17p11.2(16,774,233-20,243,349)x3
	46,XX	173 KB INTERSTITIAL DUPLICATION OF XP21.1->P21.1	Xp21.1(31,593,633-31,766,689)x3

Supplementary File S1:This Supplementary File lists the breakpoints for all the pathogenic CNVs (that are not incidental findings or NDD findings) listed in Table 2. This Table lists the ascertainment group, chromosome findings (if any), result of the microarray and specific breakpoints of the abnormality.
